# Analytical Method Validation of Gamijakyakgamchobuja-Tang (KCHO-1, Mecasin) Preparation

**DOI:** 10.1155/2019/7824146

**Published:** 2019-05-22

**Authors:** Tingting Wang, Seongjin Lee, Muhack Yang, Eunhye Cha, Jongwon Jang, Sungchul Kim

**Affiliations:** ^1^Department of Acupuncture & Moxibustion Medicine, Wonkwang University Gwangju Korean Medicine Hospital, Gwangju 61729, Republic of Korea; ^2^Nervous & Muscular System Disease Clinical Research Center of Wonkwang University Gwangju Korean Medical Hospital, Gwangju 61729, Republic of Korea

## Abstract

Previous studies have confirmed that KCHO-1 (Mecasin) was developed to alleviate the symptoms of Amyotrophic Lateral Sclerosis (ALS). And its toxicity test has also been carried out. The aim of this study is confirming the validation and stability of concentration analysis method of the Mecasin preparations using HPLC. As a conclusion, we found that the preparations at the concentrations of 50mg/ml and 200mg/ml in sterilized distilled water were homogeneous and it was stable for 4 hours at room temperature and 7 days refrigerated condition (2~8°C). And this method for analyzing the concentration of the Mecasin preparations has been found to be suitable. This study is helpful to promote development of reliable manufacturing medicine and good researches through definitive quality control of Mecasin as complex herbal medicine, aiming to provide help for the treatment of ALS.

## 1. Introduction

**Figure 1:**
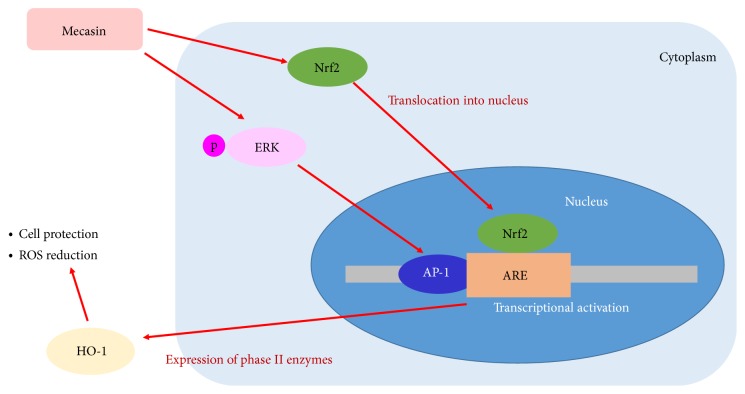
1st mechanism of action of Mecasin.

**Figure 2:**
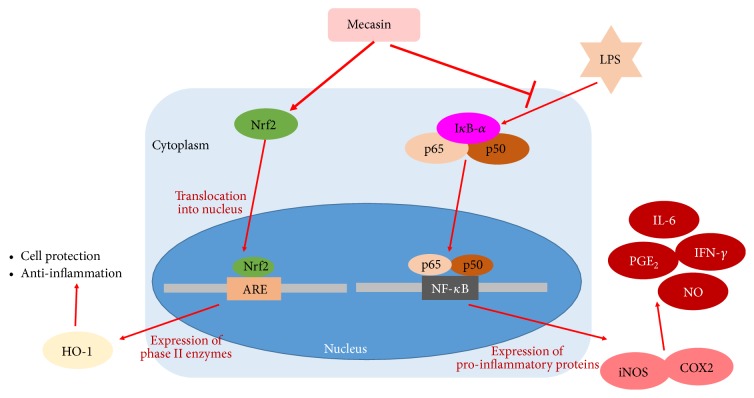
2nd mechanism of action of Mecasin.

Gamijakyakgamchobuja-tang (KCHO-1, Mecasin) is a new prescription reported to have anti-inflammatory and antioxidant properties [1]. The constituents of Mecasin are Radix Paeoniae Alba, Radix Glycyrrhizae, Radix Aconiti Lateralis Preparata, Radix Salviae Miltiorrhizae, Rhizoma Gastrodiae, Radix Polygalae, Curcuma Root, Fructus Chaenomelis, and Rhizoma Atractylodis Japonicae (Table 1) [2]. This medicine has been used mainly for alleviating pain, muscle spasms, and cold syndrome due to blood deficiency for centuries in traditional oriental medicine [3]. In recent years of medical research, we have found that it has a role in reducing pain, GABA neuron regeneration and NO reduction in neuropathic pain rats [1], antiseizure, analgesic, antipyretic, anti-inflammatory, and antiulcer effects, suppressing the progress of osteoarthritis, neuroprotective, and antineuroinflammatory effects and safety in both in vitro and in vivo trials [4–12]. More concretely, in the first mechanism of action of Mecasin, KCHO-1 increases cellular resistance to glutamate or H2O2-induced oxidative injury in HT22 cells, presumably through ERK and p38 pathways and Nrf2/ARE-dependent HO-1 expression (Figure 1) [2]. And in the second mechanism of action of Mecasin, KCHO-1 upregulated HO-1 expression by promoting the nuclear translocation of Nrf2 in mouse BV2 microglia, and it suppressed the production of proinflammatory mediators and cytokines through suppression of I*κ*B-*α* phosphorylation and degradation and NF-*κ*B nuclear translocation in LPS-stimulated microglia (Figure 2) [2, 7].

In addition, several studies have been conducted on the compositions of KCHO-1 and its toxicity. Now it is time to advance the study of KCHO-1, and the ultimate goal is to apply it to the clinic to help the treatment of ALS. And for quality control of Mecasin, confirming the validation and stability of concentration analysis method of the Mecasin preparations has become a major issue. We could promote development of reliable manufacturing medicine and good researches through definitive quality control of Mecasin as complex herbal medicine by the validation and stability studies.

The experiments for this research were conducted at the Korea Testing & Research Institute (KTR), an institution authorized to perform nonclinical studies under the GLP regulations.

## 2. Materials and Methods

### 2.1. Analytical Method Validation

*(1) Test Article and Vehicle Control*. The test article was KCHO-1, which was provided by the Nervous & Muscular System Disease Clinical Research Center of Wonkwang University Gwang-ju Korean Medicine Hospital and stored at room temperature (1~30°C). The vehicle control was sterilized distilled water manufactured by Korean Sci. Standard substances were Curcumin, Glycyrrhizic Acid, and Salvianolic Acid B. All were provided by ChromaDex, with assay of 97.7, 93.3, and 96.7%, respectively.

*(2) Reagents and Equipment*. The reagents were acetonitrile, purified water, methanol, ethanol (Burdick and Jackson, USA). Balance (KG-EQM-042) and micropipette (KG-EQM-359) were used as equipment. Shimadzu HPLC (KG-EQM-352(8)) was used as the analytical instrument and the analysis conditions were as follows (Table 2).

*(3) Preparation of Solvents and Methods*

*(i) Mobile Phase*

  A line: water
  B line: acetonitrile

The mobile phase was used within 7 days.

*(ii) Diluent*. Ethanol: methanol (50:50, v/v) was used as diluent solvent.

*(iii) Standard Solution*

*(a) Curcumin*. 20mg of the standard substance was weighed and placed in a 10mL volumetric flask, and diluted solvent was added to the line. This was used as a stock solution. The stock solution was diluted to 1, 5, 10, 50, and 100*μ*g/mL with dilution solvent and used as a standard solution.

*(b) Glycyrrhizic Acid*. 20mg of the standard substance was weighed and placed in a 10mL volumetric flask, and diluted solvent was added to the line. This was used as a stock solution. The stock solution was diluted to 5, 25, 50, 250, and 500*μ*g/mL with dilution solvent and used as a standard solution.

*(c) Salvianolic Acid B*. 20mg of the standard substance was weighed and placed in a 10mL volumetric flask, and diluted solvent was added to the line. This was used as a stock solution. The stock solution was diluted to 5, 25, 50, 250, and 500*μ*g/mL with dilution solvent and used as a standard solution.

*(4) QC Sample*

*(a) Curcumin*. A 50*μ*g/mL concentration of the standard solution was used.

*(b) Glycyrrhizic Acid*. A 250*μ*g/mL concentration of the standard solution was used.

*(c) Salvianolic Acid B*. A 250*μ*g/mL concentration of the standard solution was used.

*(5) Preparation of Test Article and Treatment of Preparation*. 1500 mg and 6000 mg of the test article were weighed and added with a vehicle (sterilized distilled water), shaken, and adjusted to 30mL. Concentrations of preparations for homogeneity and stability tests were 50mg/mL and 200mg/mL. 1mL of the preparation at a concentration of 50mg/mL and 200mg/mL was diluted with a diluting solvent and injected in 20 mL each into HPLC within the calibration curve range.

*(6) Quantitation*. The quantitative value of the preparation was calculated by the following equation after substituting the peak area of the measured value into the calibration curve (y = ax + b).

- The quantitative value of the preparation: the measured value × dilution factor

The coefficient of variation, accuracy, and rate of variation were calculated as follows.

- The coefficient of variation: (Standard deviation of quantitative values ÷ Average of quantitative values) × 100
- The accuracy: (Average of quantitative values ÷ Theoretical concentration) × 100
- The rate of variation: [(Average of Quantitative value after storage - Quantitative value average immediately after sample preparation) ÷ Quantitative value average immediately after sample preparation]) × 100

*(4) Preparation Analysis*

*(i) System Suitability*. The coefficient of variation was calculated by repeatedly measuring QC samples 5 times. The criterion was that the coefficient of variation of peak area and retention time was less than 3%.

*(ii) Linearity*. The concentration of the standard solution was measured once each time, and the correlation coefficient between the concentration of the standard solution and the peak area was calculated. The criterion was that the correlation coefficient r was 0.9950 or more. The results used for linearity validation were used as calibration curves for stability analysis.

*(iii) Specificity*. Blank samples were measured and the presence or absence of interference peaks was confirmed at the same position as the retention time of the test substance. The criterion is that the peaks of the test substance exhibit sufficient shape for quantification and that there is no interference peak at the same retention time as the test substance.

*(iv) Intraday*. The preparation was sampled three times in the middle layer and measured once per sample. The criterion was that the variation coefficient of the quantitative value was 15% or less and the accuracy was 75 to 125%.

*(v) Stability in Autosampler*. In order to confirm the stable time in the autosampler, the samples used in the intraday were left in the autosampler for a certain time and then remeasured. The criterion was that the variation coefficient of the quantitative value was 15% or less and the variation rate with respect to the initial concentration was within ±25%.

*(vi) Homogeneity*. The preparation was sampled each three times in the upper, middle, and lower layers and measured once per sample. The results of the middle layer were used as a result of intraday. The criterion was that the variation coefficient of the quantitative value was 15% or less and the accuracy was 75 to 125%.

*(vii) Stability*

*(a) Room Temperature for 4 Hours*. The preparation for each dose was left at room temperature for 4 hours, sampled three times in the middle layer, and measured once per sample; the stability was confirmed. The criterion was that the variation coefficient of the quantitative value was 15% or less and the variation rate with respect to the initial concentration was within ±25%.

*(b) Refrigeration for 7 Days*. The preparation for each dose was left at refrigerated condition (2~8°C) for 7 days, sampled three times in the middle layer, and measured once per sample; the stability was confirmed. The criterion was that the variation coefficient of the quantitative value was 15% or less and the variation rate with respect to the initial concentration was within ±25%.

*(8) QC (Quality Control)*. The qc samples were measured three times at the end of the analysis. The criterion was that the coefficient of variation of the analysis result was less than 10% and the accuracy was 80~120%.

## 3. Results

### 3.1. Analytical Method Validation

*(1) System Suitability*. The coefficient of variation of the peak area and retention time measured five times repeatedly at a concentration of 50*μ*g/ mL of the QC sample of the Curcumin was 0.20% and 0.03%.

The coefficient of variation of the peak area and retention time measured five times repeatedly at a concentration of 250*μ*g/ mL of the QC sample of the Glycyrrhizic Acid was 0.32% and 0.04%.

The coefficient of variation of the peak area and retention time measured five times repeatedly at a concentration of 250*μ*g/ mL of the QC sample of the Salvianolic Acid B was 0.37% and 0.02%. The results are shown in Table 3.

*(2) Linearity*. The correlation coefficient *r* of the calibration curve measured at the concentration range of 1 to 100 *μ*g /mL of the standard solution of the Curcumin was 1.0000 on day 0 and 0.9999 on day 7.

The correlation coefficient *r* of the calibration curve measured at the concentration range of 5 to 500 *μ*g /mL of the standard solution of the Glycyrrhizic Acid was 1.0000 on day 0 and 1.0000 on day 7.

The correlation coefficient *r* of the calibration curve measured at the concentration range of 5 to 500 *μ*g /mL of the standard solution of the Salvianolic Acid B was 0.9998 on day 0 and 1.0000 on day 7. The results are shown in Tables 4 and 5.

**Figure 3:**
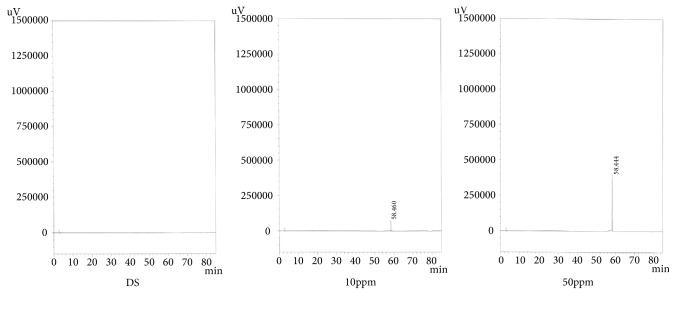
Chromatogram of specificity (Curcumin).

**Figure 4:**
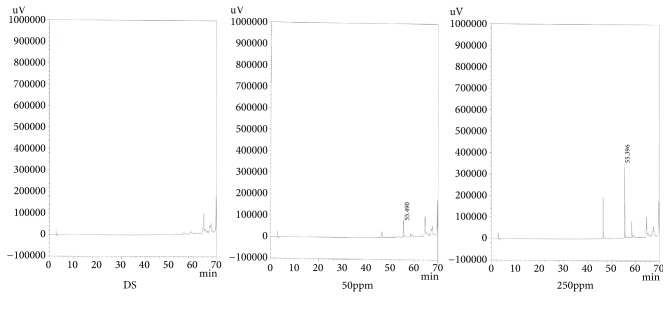
Chromatogram of specificity (Glycyrrhizic Acid).

**Figure 5:**
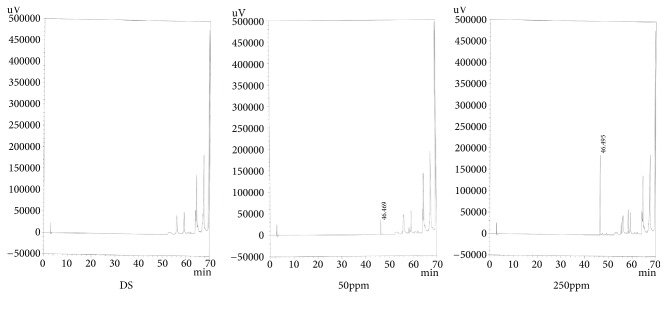
Chromatogram of specificity (Salvianolic Acid B).

*(3) Specificity*. The preparation exhibited sufficient shape for analysis and no component that affected the peak of the test substance during the analysis was detected. The results are shown in Figures 3, 4, and 5.

*(4) Intraday*. The coefficient of variation of the test substance was 0.82% and 1.66% and the accuracy was 116.99% and 116.32% at a concentration of Curcumin of 50mg/mL and 200mg/mL of the preparation by each dose.

The coefficient of variation of the test substance was 2.07% and 0.64% and the accuracy was 78.64% and 79.02% at a concentration of Glycyrrhizic Acid of 50mg/mL and 200mg/mL of the preparation by each dose.

The coefficient of variation of the test substance was 0.86% and 1.30% and the accuracy was 95.61% and 94.70% at a concentration of Salvianolic Acid B of 50mg/mL and 200mg/mL of the preparation by each dose. The results are shown in Table 6.

*(5) Stability in Autosampler*. As a result of confirming the stability of the Curcumin concentration in the autosampler at 50mg/mL and 200mg/mL for each dose, the variation rates with respect to the initial concentration were 1.15% and 0.85%, and the coefficient of variation was 0.59% and 0.88%.

As a result of confirming the stability of the Glycyrrhizic Acid concentration in the autosampler at 50mg/mL and 200mg/mL for each dose, the variation rates with respect to the initial concentration were 0.99% and 1.85%, and the coefficient of variation was 0.42% and 0.95%.

As a result of confirming the stability of the Salvianolic Acid B concentration in the autosampler at 50mg/mL and 200mg/mL for each dose, the variation rates with respect to the initial concentration were 1.31% and 0.22%, and the coefficient of variation was 1.08% and 0.45%. The results are shown in Table 7.

*(6) Homogeneity*. The homogeneity of the upper, middle, and lower layers at the concentrations of Curcumin at 50mg/mL and 200mg/mL of the preparations was confirmed. The coefficient of variation was 2.52% and 1.73%, and the accuracy was 117.65% and 115.91%.

The homogeneity of the upper, middle, and lower layers at the concentrations of Glycyrrhizic Acid at 50mg/mL and 200mg/mL of the preparations was confirmed. The coefficient of variation was 3.35% and 2.08%, and the accuracy was 81.53% and 78.97%.

The homogeneity of the upper, middle, and lower layers at the concentrations of Salvianolic Acid B at 50mg/mL and 200mg/mL of the preparations was confirmed. The coefficient of variation was 2.39% and 1.40%, and the accuracy was 97.49% and 95.17%. The results are shown in Table 8.

*(7) Stability*

*(i) Room Temperature for 4 Hours*. As a result of confirming the stability of the concentration of Curcumin at 50mg/mL and 200mg/mL of the preparation at room temperature for 4 hours, the variation rates with respect to the initial concentration immediately after preparation was -1.04% and -0.39%, and the coefficient of variation was 1.66% and 2.78%.

As a result of confirming the stability of the concentration of Glycyrrhizic Acid at 50mg/mL and 200mg/mL of the preparation at room temperature for 4 hours, the variation rates with respect to the initial concentration immediately after preparation were 1.19% and -0.76%, and the coefficient of variation was 4.36% and 1.49%.

As a result of confirming the stability of the concentration of Salvianolic Acid B at 50mg/mL and 200mg/mL of the preparation at room temperature for 4 hours, the variation rates with respect to the initial concentration immediately after preparation were -0.30% and 0.52%, and the coefficient of variation was 2.69% and 1.63%. The results are shown in Table 9.

*(ii) Refrigeration for 7 Days*. As a result of confirming the stability of the concentration of Curcumin at 50mg/mL and 200mg/mL of the preparation at refrigerated condition (2~8°C) for 7 days, the variation rates with respect to the initial concentration immediately after preparation were -18.39% and -18.53%, and the coefficient of variation was 2.80% and 3.37%.

As a result of confirming the stability of the concentration of Glycyrrhizic Acid at 50mg/mL and 200mg/mL of the preparation at refrigerated condition (2~8°C) for 7 days, the variation rates with respect to the initial concentration immediately after preparation were 13.18% and 17.02%, and the coefficient of variation was 2.48% and 2.99%.

As a result of confirming the stability of the concentration of Salvianolic Acid B at 50mg/mL and 200mg/mL of the preparation at refrigerated condition (2~8°C) for 7 days, the variation rates with respect to the initial concentration immediately after preparation were 4.18% and 7.01%, and the coefficient of variation was 2.26% and 2.91%. The results are shown in Table 10.

*(8) QC (Quality Control)*. When the concentration of 50*μ*g/mL of the QC sample of Curcumin was measured three times at the end of the analysis, the coefficient of variation was 0.41% and the accuracy was 101.78%.

When the concentration of 250*μ*g/mL of the QC sample of Glycyrrhizic Acid was measured three times at the end of the analysis, the coefficient of variation was 0.46% and the accuracy was 101.21%.

When the concentration of 250*μ*g/mL of the QC sample of Salvianolic Acid B was measured three times at the end of the analysis, the coefficient of variation was 0.39% and the accuracy was 96.92%. The results are shown in Table 11.

## 4. Discussion

Validation was performed to quantitate the concentration of the preparation to be used in the efficiency and toxicity test. As a result of the validation analysis, the peak area and the coefficient of variation of the retention time, which were measured QC samples 5 times repeatedly, satisfied all of the criteria. The linearity measured in the concentration range of the standard solution also satisfied criteria of both the correlation coefficient and the accuracy. The peak of the preparation showed a sufficient shape for analysis, and no ingredient that affected the peak of the test article was detected in the blank sample. As a result of intraday, the coefficient of variation and accuracy of the test articles in the preparations at concentrations of 50mg/mL and 200mg/mL satisfied all the criteria. 50mg/mL and 200mg/mL of the preparation were allowed to left in autosampler for a certain time and then their stability was confirmed. As a result, the variation rate and the coefficient of variation for the initial concentration of the test article for 5 hours satisfied all the criteria. The homogeneity of the upper, middle, and lower layers in the preparations at concentrations of 50mg/mL and 200mg/mL was checked. The coefficient of variation and accuracy were all satisfied the criteria. To confirm the stability, the preparation at 50mg/mL and 200mg/mL was maintained at room temperature for 4 hours and at refrigerated condition (2~8°C) for 7 days. The variation rate of the initial concentration immediately after preparation and the coefficient of variation were all satisfied with the criterion. In addition, the coefficient of variation and accuracy at the QC sample concentration satisfied all the criteria.

## 5. Conclusion

This method for analyzing the concentration of KCHO-1 preparations has been found to be suitable. The preparations at the concentrations of 50mg/ml and 200mg/ml in sterilized distilled water were homogeneous and it was stable for 4 hours at room temperature and 7-day refrigerated condition (2~8°C).

## Figures and Tables

**Table 1:** The constituents of Mecasin.

	Scientific Names	Parts
1	*Curcuma longa *	Radix

2	*Salvia miltiorrhiza *	Radix

3	*Gastrodia elata *	Rhizoma

4	*Pseudocydonia sinensis*	Fructus

5	*Paeonia lactiflora*	Radix

6	*Polygala tenuifolia*	Radix

7	*Glycyrrhiza uralensis *	Radix

8	*Atractylodes japonica *	Rhizoma

9	*Aconitum carmichaeli *	Radix Preparata

**Table 2:** Analytical conditions of KCHO-1 using HPLC.

Mobile phase	Time (min)	Water (%)	Acetonitrile (%)
	0	90	10
	30	90	10
	70	13	87
	72	13	87
	73	90	10
	85	90	10

Flow rate	1.0 mL/min		

Injection volume	20 *μ*L		

Column	CAPCELLPAK 5 C18, 250 × 2.6 mm, 5 *μ*m		

Column oven temperature	40°C		

Detector wavelength (PDA)	254 (Glycyrrhizic acid), 280 (Salvianolic acid B), 420 (Curcumin) nm		

Run time	85 min		

**Table:** (a) Curcumin

Concentration of standard solution (*μ*g/mL)	Classification	No.1	No.2	No.3	No.4	No.5	Mean	SD	CV (%)
50	Peak area	4524661	4514684	4516341	4517396	4499824	4514581	9089	0.20
	R/Time	58.43	58.42	58.41	58.45	58.41	58.43	0.02	0.03

**Table:** (b) Glycyrrhizic Acid

Concentration of standard solution (*μ*g/mL)	Classification	No.1	No.2	No.3	No.4	No.5	Mean	SD	CV (%)
50	Peak area	3923859	3913992	3910725	3923579	3893235	3913078	12516	0.32
	R/Time	55.37	55.36	55.35	55.39	55.35	55.36	0.02	0.04

**Table:** (c) Salvianolic Acid B

Concentration of standard solution (*μ*g/mL)	Classification	No.1	No.2	No.3	No.4	No.5	Mean	SD	CV (%)
50	Peak area	2079298	2079339	2082767	2064939	2068935	2075056	7675	0.37
	R/Time	46.47	46.46	46.45	46.48	46.45	46.46	0.01	0.02

**Table:** (a) Curcumin

No.	Concentration of standard solution (*μ*g/mL)	Peak Area	Measured concentration (*μ*g/mL)
1	1	85263.00	1.15

2	5	433268.00	4.97

3	10	888555.00	9.98

4	50	4511765.00	49.81

5	100	9086517.00	100.10

y=90965.1551x − 18969.5488, r=1.0000

**Table:** (b) Glycyrrhizic Acid

No.	Concentration of standard solution (*μ*g/mL)	Peak Area	Measured concentration (*μ*g/mL)
1	5	78714.00	5.30

2	25	382923.00	24.61

3	50	788267.00	50.33

4	250	3928115.00	249.56

5	500	7878090.00	500.20

y=15759.4853x − 4852.7604, r=1.0000

**Table:** (c) Salvianolic Acid B

No.	Concentration of standard solution (*μ*g/mL)	Peak Area	Measured concentration (*μ*g/mL)
1	5	32793.00	8.51

2	25	177058.00	25.17

3	50	384874.00	49.17

4	250	2073814.00	244.24

5	500	4313601.00	502.92

y=8658.3059x − 40850.7837, r=0.9998

**Table:** (a) Curcumin

No.	Concentration of standard solution	Peak Area	Measured concentration
	(*μ*g/mL)		(*μ*g/mL)
1	1	99129.00	0.66

2	5	573255.00	4.97

3	10	1110862.00	9.85

4	50	5642990.00	51.01

5	100	10983038.00	99.51

y=110099.9926x + 26535.0464, r=0.9999

**Table:** (b) Glycyrrhizic Acid

No.	Concentration of standard solution	Peak Area	Measured concentration
	(*μ*g/mL)		(*μ*g/mL)
1	5	70795.00	5.45

2	25	361460.00	25.03

3	50	736343.00	50.29

4	250	3679238.00	248.53

5	500	7422854.00	500.70

y=14845.4655x – 10159.4707, r=1.0000

**Table:** (c) Salvianolic Acid B

No.	Concentration of standard solution	Peak Area	Measured concentration
	(*μ*g/mL)		(*μ*g/mL)
1	5	36633.00	6.23

2	25	197146.00	25.21

3	50	409263.00	50.30

4	250	2069324.00	246.63

5	500	4225420.00	501.63

y=8455.2959x – 16021.9262, r=1.0000

**Table:** (a) Curcumin

Concentration of dosing formulation (*μ*g/mL)	Measured concentration (*μ*g/mL)			Mean (*μ*g/mL)	SD	CV (%)	Accuracy (%)
	No.1	No.2	No.3				
39.5	46.52	45.79	46.33	46.21	0.38	0.82	116.99
158.0	186.52	180.49	184.37	183.79	3.06	1.66	116.32

**Table:** (b) Glycyrrhizic Acid

Concentration of dosing formulation (*μ*g/mL)	Measured concentration (*μ*g/mL)			Mean (*μ*g/mL)	SD	CV (%)	Accuracy (%)
	No.1	No.2	No.3				
305.0	234.75	240.17	244.67	239.86	4.97	2.07	78.64
1220.0	961.40	959.78	971.12	964.10	6.13	0.64	79.02

**Table:** (c) Salvianolic Acid B

Concentration of dosing formulation (*μ*g/mL)	Measured concentration (*μ*g/mL)			Mean (*μ*g/mL)	SD	CV (%)	Accuracy (%)
	No.1	No.2	No.3				
985.0	932.67	944.18	948.37	941.74	8.13	0.86	95.61
3940.0	3762.21	3675.21	3755.96	3731.13	48.53	1.30	94.70

**Table:** (a) Curcumin

Title	Concentration of the dosing formulation (*μ*g/mL)	Measured concentration			Mean (*μ*g/mL)	SD	CV (%)	Accuracy (%)	Variation (%)
		(*μ*g/mL)							
		No.1	No.2	No.3					
Start	39.5	46.52	45.79	46.33	46.21	0.38	0.82	116.99	-
	158.0	186.52	180.49	184.37	183.79	3.06	1.66	116.32	-

End	39.5	47.06	46.57	46.59	46.74	0.28	0.59	118.33	1.15
	158.0	186.74	183.55	185.76	185.35	1.63	0.88	117.31	0.85

**Table:** (b) Glycyrrhizic Acid

Title	Concentration of the dosing formulation (*μ*g/mL)	Measured concentration			Mean (*μ*g/mL)	SD	CV (%)	Accuracy (%)	Variation (%)
		(*μ*g/mL)							
		No.1	No.2	No.3					
Start	305.0	234.75	240.17	244.67	239.86	4.97	2.07	78.64	-
	1220.0	961.40	959.78	971.12	964.10	6.13	0.64	79.02	-

End	305.0	241.22	242.24	243.27	242.24	1.03	0.42	79.42	0.99
	1220.0	943.30	956.29	939.06	946.22	8.98	0.95	77.56	-1.85

**Table:** (c) Salvianolic Acid B

Title	Concentration of the dosing formulation (*μ*g/mL)	Measured concentration			Mean (*μ*g/mL)	SD	CV (%)	Accuracy (%)	Variation (%)
		(*μ*g/mL)							
		No.1	No.2	No.3					
Start	985.0	932.67	944.18	948.37	941.74	8.13	0.86	95.61	-
	3940.0	3762.21	3675.21	3755.96	3731.13	48.53	1.30	94.70	-

End	985.0	945.25	965.36	951.74	954.12	10.26	1.08	96.86	1.31
	3940.0	3758.11	3726.34	3733.48	3739.31	16.67	0.45	94.91	0.22

**Table:** (a) Curcumin

Concentration of dosing formulation (*μ*g/mL)		Measured concentration (*μ*g/mL)			Mean (*μ*g/mL)	SD	CV (%)	Accuracy (%)
		No.1	No.2	No.3				
39.5	Upper	46.67	47.97	46.87	46.47	1.17	2.52	117.65
	Middle	46.66	46.08	46.08				
	Lower	43.99	47.82	46.06				

158.0	Upper	177.77	182.59	187.16	183.13	3.17	1.73	115.91
	Middle	186.58	182.00	184.79				
	Lower	183.97	179.00	184.33				

**Table:** (b) Glycyrrhizic Acid

Concentration of dosing formulation (*μ*g/mL)		Measured concentration (*μ*g/mL)			Mean (*μ*g/mL)	SD	CV (%)	Accuracy (%)
		No.1	No.2	No.3				
305.0	Upper	252.52	260.77	261.73	248.68	8.32	3.35	81.53
	Middle	238.85	248.52	245.37				
	Lower	243.74	247.37	239.27				

1220.0	Upper	975.53	947.51	962.39	963.45	20.06	2.08	78.97
	Middle	938.64	955.67	954.80				
	Lower	951.83	980.93	1003.77				

**Table:** (c) Salvianolic Acid B

Concentration of dosing formulation (*μ*g/mL)		Measured concentration (*μ*g/mL)			Mean (*μ*g/mL)	SD	CV (%)	Accuracy (%)
		No.1	No.2	No.3				
985.0	Upper	966.20	1001.76	988.87	960.32	22.91	2.39	97.49
	Middle	934.32	958.07	945.25				
	Lower	938.01	964.54	945.84				

3940.0	Upper	3739.49	3721.20	3813.87	3749.74	52.53	1.40	95.17
	Middle	3759.39	3704.46	3731.31				
	Lower	3694.78	3728.22	3854.97				

**Table:** (a) Curcumin

Time (hr)	Concentration of the dosing formulation (*μ*g/mL)	Measured concentration			Mean (*μ*g/mL)	SD	CV (%)	Accuracy (%)	Variation (%)
		(*μ*g/mL)							
		No.1	No.2	No.3					
0	39.5	46.52	45.79	46.33	46.21	0.38	0.82	116.99	-
	158.0	186.52	180.49	184.37	183.79	3.06	1.66	116.32	-

4	39.5	46.37	45.93	44.89	45.73	0.76	1.66	115.77	-1.04
	158.0	178.89	181.59	188.74	183.07	5.09	2.78	115.87	-0.39

**Table:** (b) Glycyrrhizic Acid

Time (hr)	Concentration of the dosing formulation (*μ*g/mL)	Measured concentration			Mean (*μ*g/mL)	SD	CV (%)	Accuracy (%)	Variation (%)
		(*μ*g/mL)							
		No.1	No.2	No.3					
0	305.0	234.75	240.17	244.67	239.86	4.97	2.07	78.64	-
	1220.0	961.40	959.78	971.12	964.10	6.13	0.64	79.02	-

4	305.0	254.16	240.70	233.31	242.72	10.57	4.36	79.58	1.19
	1220.0	947.84	973.20	949.28	956.77	14.24	1.49	78.42	-0.76

**Table:** (c) Salvianolic Acid B

Time (hr)	Concentration of the dosing formulation (*μ*g/mL)	Measured concentration			Mean (*μ*g/mL)	SD	CV (%)	Accuracy (%)	Variation (%)
		(*μ*g/mL)							
		No.1	No.2	No.3					
0	985.0	932.67	944.18	948.37	941.74	8.13	0.86	95.61	-
	3940.0	3762.21	3675.21	3755.96	3731.13	48.53	1.30	94.70	-

4	985.0	964.42	938.51	913.89	938.94	25.27	2.69	95.32	-0.30
	3940.0	3680.07	3779.39	3791.90	3750.45	61.27	1.63	95.19	0.52

**Table:** (a) Curcumin

Day	Concentration of the dosing formulation (*μ*g/mL)	Measured concentration			Mean (*μ*g/mL)	SD	CV (%)	Accuracy (%)	Variation (%)
		(*μ*g/mL)							
		No.1	No.2	No.3					
0	39.5	46.52	45.79	46.33	46.21	0.38	0.82	116.99	-
	158.0	186.52	180.49	184.37	183.79	3.06	1.66	116.32	-

7	39.5	37.92	38.65	36.57	37.71	1.06	2.80	95.48	-18.39
	158.0	143.93	152.77	152.53	149.74	5.04	3.37	94.77	-18.53

**Table:** (b) Glycyrrhizic Acid

Day	Concentration of the dosing formulation (*μ*g/mL)	Measured concentration			Mean (*μ*g/mL)	SD	CV (%)	Accuracy (%)	Variation (%)
		(*μ*g/mL)							
		No.1	No.2	No.3					
0	305.0	234.75	240.17	244.67	239.86	4.97	2.07	78.64	-
	1220.0	961.40	959.78	971.12	964.10	6.13	0.64	79.02	-

7	305.0	277.24	273.06	264.09	271.46	6.72	2.48	89.00	13.18
	1220.0	1099.67	1165.43	1119.48	1128.19	33.73	2.99	92.47	17.02

**Table:** (c) Salvianolic Acid B

Day	Concentration of the dosing formulation (*μ*g/mL)	Measured concentration			Mean (*μ*g/mL)	SD	CV (%)	Accuracy (%)	Variation (%)
		(*μ*g/mL)							
		No.1	No.2	No.3					
0	985.0	932.67	944.18	948.37	941.74	8.13	0.86	95.61	-
	3940.0	3762.21	3675.21	3755.96	3731.13	48.53	1.30	94.70	-

7	985.0	996.37	991.24	955.72	981.11	22.14	2.26	99.61	4.18
	3940.0	3879.51	4111.82	3986.46	3992.60	116.28	2.91	101.34	7.01

**Table:** (a) Curcumin

Concentration of total catechins (*μ*g/mL)	Measured concentration (*μ*g/mL)			Mean (*μ*g/mL)	SD	CV (%)	Accuracy (%)
	No.1	No.2	No.3				
50	50.66	51.07	50.93	50.89	0.21	0.41	101.78

**Table:** (b) Glycyrrhizic Acid

Concentration of total catechins (*μ*g/mL)	Measured concentration (*μ*g/mL)			Mean (*μ*g/mL)	SD	CV (%)	Accuracy (%)
	No.1	No.2	No.3				
250	251.68	253.84	253.55	253.02	1.17	0.46	101.21

**Table:** (c) Salvianolic Acid B

Concentration of total catechins (*μ*g/mL)	Measured concentration (*μ*g/mL)			Mean (*μ*g/mL)	SD	CV (%)	Accuracy (%)
	No.1	No.2	No.3				
250	242.70	242.95	241.21	242.29	0.94	0.39	96.92

## Data Availability

The data used to support the findings of this study are available from the corresponding author upon request.

## References

[1] Kim D. H. (2015). *Effect of Gamijakyakgamchobuja-Tang on Neuropathic Pain in Rats*.

[2] Lee D.-S., Ko W., Song B.-K. (2016). The herbal extract KCHO-1 exerts a neuroprotective effect by ameliorating oxidative stress via heme oxygenase-1 upregulation. *Molecular Medicine Reports*.

[3] Guo L., Cho S. Y., Kang S. S., Lee S. H., Baek H. Y., Kim Y. S. (2007). Orthogonal array design for optimizing extraction efficiency of active constituents from Jakyak-Gamcho Decoction, the complex formula of herbal medicines, Paeoniae Radix and Glycyrrhizae Radix. *Journal of Ethnopharmacology*.

[4] Yu J. W. (2010). *Study on the Ingredient of Jakyakgamchotang*.

[5] Kim B. W. (2010). Anti-inflammatory effect of Jakyakgamcho-tang. *The Korean Journal of Internal Medicine*.

[6] Lee J. M., Hong S. Y., Oh M. S. (2013). Effects of Jakyakkamchobuja-tang on Papain-induced osteoarthritis in mice. *Journal of Korean Oriental Medicine*.

[7] Lee D.-S., Ko W., Yoon C.-S. (2014). KCHO-1, a novel anti-neuroinflammatory agent, inhibits lipopolysaccharide-induced neuroinflammatory responses through Nrf2-mediated heme oxygenase-1 expression in mouse BV2 microglia cells. *Evidence-Based Complementary and Alternative Medicine*.

[8] Cha E., Lee J., Lee S. (2015). A 4-week repeated dose oral toxicity study of Mecasin in Sprague-Dawley rats to determine the appropriate doses for a 13-week, repeated toxicity test. *Journal of Pharmacopuncture*.

[9] Jeong H., Lee J., Cha E. (2014). A study on the oral toxicity of mecasin in rats. *Journal of Pharmacopuncture*.

[10] Cha E., Jeong H., Lee J., Lee S., Park M., Kim S. (2015). A study on single dose toxicity of mecasin pharmacopuncture injection in muscle. *Journal of Korean Medicine*.

[11] Lee S. J., Jeong H. H., Lee J. C. (2016). A study on single dose toxicity of intravenous injection of mecasin herbal acupuncture. *The Acupuncture*.

[12] Kook M. G., Choi S. W., Seo Y. (2017). KCHO-1, a novel herbal anti-inflammatory compound, attenuates oxidative stress in an animal model of amyotrophic lateral sclerosis. *Journal of Veterinary Science*.

